# Proportion of Adults Who Identified Walking As a US Surgeon General Priority

**DOI:** 10.5888/pcd15.170417

**Published:** 2018-05-24

**Authors:** Gayathri Suresh Kumar, Kathleen B. Watson, David R. Brown, Susan A. Carlson

**Affiliations:** 1 Division of Nutrition, Physical Activity, and Obesity, National Center for Chronic Disease Prevention and Health Promotion, Centers for Disease Control and Prevention, Atlanta, Georgia

## Abstract

In September 2015, Step It Up! The Surgeon General’s Call to Action to Promote Walking and Walkable Communities (Call to Action) was released. This descriptive study reports the proportion of adults who responded to the 2016 Summer ConsumerStyles survey (N = 4,114) who identified walking as the activity the US Surgeon General recently promoted in the Call to Action to help Americans be more physically active. Less than half of adults (44%) correctly identified walking. Adults who were aged 18 to 24 years (35%), were male (43%), were non-Hispanic white (42%), or were physically inactive (36%) were less likely to identify walking than their counterparts. This study highlights an opportunity to raise awareness and promote the Call to Action, especially among certain populations.

## Objective

In September 2015, Step It Up! The Surgeon General’s Call to Action to Promote Walking and Walkable Communities (Call to Action) was released (1). In this report, the US Surgeon General called on Americans to be more physically active through increasing walking behaviors and the number of walkable communities (1). However, whether people are aware of this call is unknown. As an indicator of awareness, we assessed the proportion of US adults who correctly identified walking as the activity the Surgeon General recently highlighted to increase physical activity and how they heard about this activity. To our knowledge, this is the first time information about awareness of the Surgeon General’s call has been reported. This information can assist in determining the reach of the call as well as if continued promotional efforts are needed and among whom they are needed.

## Methods

Data were licensed from Porter Novelli’s 2016 Summer ConsumerStyles survey, an annual consumer online panel survey (response rate = 68%: 4,203 respondents of 6,166 panelists surveyed), which was fielded from June 24, 2016, to July 11, 2016 (2). Respondents were asked “What activity has the Surgeon General recently promoted to help Americans be more physically active?” Respondents were classified into 2 groups: 1) adults who correctly identified walking and 2) adults who identified another activity (sports, bicycling, dancing, none of the above) or who responded “don’t know/not sure.” To assess the medium through which respondents heard about the activity, respondents were asked, “How did you hear about what the Surgeon General recently promoted to help Americans be more physical active?” Response options included: news outlet (television/radio); news outlet (print or online); internet; social media; friends/family; none of these; “I did not hear anything”; and don’t know/not sure. Multiple sources could be selected. Respondents were categorized as they did not hear anything or they heard something (all other responses).

We excluded 89 respondents with missing data on sociodemographic characteristics, physical activity level, activity recently promoted, or medium via which they heard about the activity. Analyses were exempt from the Centers for Disease Control and Prevention’s institutional review board process because data were de-identified.

The percentage of adults who correctly identified walking was examined by sociodemographic characteristics and physical activity level. Contrasts were used to identify significant (*P* < .05) trends and differences. Among respondents who correctly identified walking as the activity, we examined how respondents heard about the activity. Data were weighted to match the 2015 US Current Population Survey proportions for sex, age, household income, education level, race/ethnicity, household size, metropolitan statistical area status, census region, and whether a respondent had internet access before joining the panel. Analyses incorporated sample weights and were performed using SAS version 9.3 (SAS Institute, Inc).

## Results

Overall, 44.4% of adults identified walking as the activity recently promoted by the Surgeon General to increase physical activity (Table). Respondents who indicated that they didn’t know about the activity was selected by 48.0% of respondents, and 7.6% selected another activity or none. Compared with non-Hispanic black and Hispanic adults, a significantly lower proportion of non-Hispanic white adults and adults of other race/ethnicities identified walking. A significantly lower proportion of men than women identified walking as the behavior. The proportion of people who correctly identified walking increased with increasing age and physical activity level (test of linear trend; *P* < .05). 

**Table:** Sample Description and Proportion of Respondents Who Identified Walking as the Activity the US Surgeon General Recently Promoted to Help Adults be More Physically Active, Summer ConsumerStyles, 2016^a^

Characteristic	Unweighted Sample Size (Weighted %)^a^	Percentage of Respondents Who Identified Walking^b^ (95% Confidence Interval)
**Total**	4,114 (100)	44.4 (42.6–46.2)
**Sex**		
Male	1,959 (48.4)	42.5^c^ (39.9–45.1)
Female	2,155 (51.6)	46.2^d^ (43.6–48.7)
**Age, y**		
18–24	258 (12.1)	35.2^c^ (28.8–41.5)
25–44	1,190 (34.1)	40.5^c^ (37.2–43.8)
45–64	1,736 (34.8)	48.0^d^ (45.2–50.8)
≥65	930 (19.0)	50.7^d^ (46.9–54.5)
**Race/ethnicity**		
White, non-Hispanic	3,043 (65.1)	41.8^c^ (39.7–43.8)
Black, non-Hispanic	417 (11.7)	52.0^d^ (46.3–57.8)
Hispanic	455 (15.2)	51.3^d^ (45.9–56.7)
Other, multiracial	199 (7.9)	41.1^c^ (32.9–49.4)
**Education level**		
<High school graduate	265 (11.8)	46.7 (39.9–53.5)
High school graduate	1,214 (29.5)	41.3 (38.1–44.5)
Some college	1,251 (28.5)	46.8 (43.4–50.1)
College graduate	1,384 (30.2)	44.3 (41.2–47.5)
**Household income, $**		
<24,999	700 (17.2)	43.9 (39.3–48.5)
25,000–39,999	660 (13.0)	44.8 (40.1–49.4)
40,000–59,999	676 (15.9)	45.1 (40.5–49.6)
≥60,000	2,078 (53.9)	44.2 (41.7–46.8)
**Physical activity level^e^^,^ f**		
Inactive	617 (14.7)	36.4^c^ (31.9–40.8)
Insufficiently active	1,099 (26.9)	43.7^d^ (40.2–47.3)
Active	2,398 (58.4)	46.7^d^ (44.3–49.1)

a Sample percentages may not add to 100% because of rounding. Data were weighted to match the 2015 US Current Population Survey proportions for sex, age, household income, education level, race/ethnicity, household size, metropolitan statistical area status, census region, and whether a respondent had internet access before joining the panel.

b Selected walking for the response to “What activity has the Surgeon General recently promoted to help Americans be more physically active?” Proportion of respondents who selected other responses to this question include: 1.7% (sports); 2.3% (bicycling); 2.5% (dancing); 1.1% (none); and 48.0% (don’t know).

c Significant at *P* < .05. Significant pairwise comparison where values denoted by footnote c are less than the values denoted by footnote d.

d Significant at *P* < .05. Significant pairwise comparison where values denoted by footnote c are less than the values denoted by footnote d.

e Physical activity level assessed by 4 questions: a) In a usual week, how many days do you do vigorous leisure-time physical activities for at least 10 minutes that cause heavy sweating or large increases in breathing or heart rate? b) About how long do you do these vigorous leisure-time physical activities each day? c) In a usual week, how many days do you do moderate leisure-time physical activities for at least 10 minutes that cause only medium sweating or a moderate increase in breathing or heart rate? and d) About how long do you do these moderate leisure-time physical activities each day?

f Physical activity level definitions: active (meeting the aerobic physical activity guideline of 150 min/week of moderate-intensity physical activity, 75 min/week of vigorous-intensity physical activity, or an equivalent combination) (7); insufficiently active (some activity, but not enough to meet active definition); and inactive (no leisure-time physical activity for at least 10 min) (7).

Among adults identifying walking as the activity, 72.5% reported the source by which they heard about the activity (Figure). Among these adults, 37.7% reported that they heard about the activity via news outlet on television or radio, and 37.8% did not know or were not sure how they heard about the activity. Among all adults, 32.2% identified walking as the activity and reported a source from where they heard this information.

**Figure:**
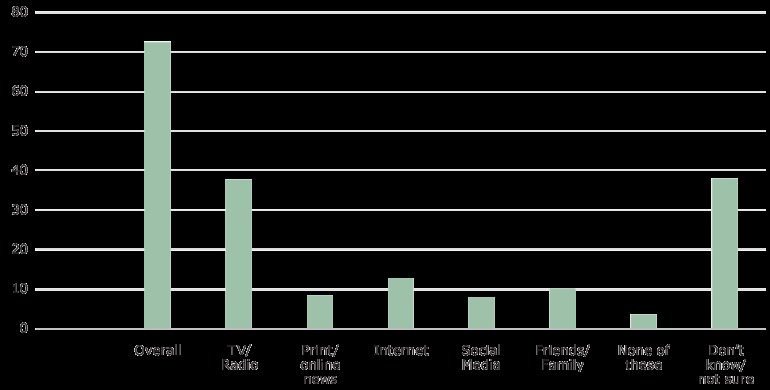
Percentage of Respondents Who Were Aware That Walking Was the Activity Recently Promoted by the Surgeon General to Increase Physical Activity and the Medium From Which They Received the Information, Summer ConsumerStyles, 2016. Values do not total 100% because respondents could choose more than one response. Percentages based on the 72.5% of respondents who heard something about the activity. **Table:** 

Medium	Heard Something About the Activity, %
Overall	72.5
Television/radio	37.7
Print/online news	8.3
Internet	12.7
Social media	7.9
Friends/family	9.9
None of these	3.6
Don’t know/not sure	37.8

## Discussion

Less than half (44%) of US adults correctly identified walking as the activity the Surgeon General recently promoted to help Americans be more physically active. Adults who were younger, male, or white or other racial/ethnic group or who were physically inactive were less likely to identify walking than their counterparts. More work is needed to communicate the Call to Action, especially among the demographic groups who were less likely to identify walking.

Among those who reported that walking was the activity being promoted, the most common way of hearing about the activity was via news outlets on television or radio. However, during the first month following the Call to Action’s release, 2,080 stories appeared in traditional media outlets; most stories appeared on news web sites (n = 1,036) or in print (n = 753), with much fewer appearing on television (n = 225) and radio (n = 24) (3). Despite fewer stories via television and radio, most Americans still receive their news from television (57%) (4), which could explain why more respondents reported hearing about the activity via television or radio.

Differences by demographic characteristics in correctly identifying walking were observed, some of which may be explained by different patterns of media use. For example, we found that younger adults were less likely to identify walking, which could be because they spend less time consuming news (4). We also found that black adults were more likely to identify walking than white adults; higher consumption of print media (5) and television by blacks compared with the general population and high use (56 hours per month) of mobile app or internet browsers (6) by blacks may have increased their exposure to the Call to Action.

The Call to Action calls on Americans to be more physically active through improved walking and walkability. We used a respondent’s ability to identify walking as the activity as an indicator of whether adults were aware of the Call to Action; however, this type of proxy may have overestimated awareness. Our finding that 27% of those correctly identifying walking reported that they did not hear anything highlights this concern. When these respondents were excluded, the proportion correctly identifying walking was 32.2%.

Study limitations were the use of an online panel survey, which may not be representative of the national population. Furthermore, because the survey was fielded almost one year after the release, respondents may not recall where they heard about the call, which may be why many selected don’t know/not sure. It is also possible that some respondents who selected don’t know/not sure may not have heard about the call.

Several federal and nonfederal partners have taken action to increase awareness and promote walking and walkable communities (3). This study highlights the need for continued promotion of messages from the Call to Action. A targeted social media approach for specific demographic populations, such as the use of Snapchat and Instagram for younger age groups, can help promote the messages among these populations (8).
